# Juvenile Justice-Translating Research Interventions for Adolescents in the Legal System (JJ-TRIALS): a multi-site, cooperative implementation science cooperative

**DOI:** 10.1186/1748-5908-10-S1-A43

**Published:** 2015-08-20

**Authors:** Tisha Wiley, Steven Belenko, Knight, Danica, John Bartkowski, Angela Robertson, Gregory Aarons, Gail Wasserman, Carl Leukefeld, Ralph DiClemente, Dionne Jones

**Affiliations:** 1 National Institute on Drug Abuse, Bethesda, MD 20852, USA; 2 Temple University, Philadelphia, PA 19122, USA; 3 Texas Christian University, Fort Worth, TX 76129, USA; 4 University of Texas at San Antonio, San Antonio, TX 78249 USA; 5 Mississippi State University, Mississippi State, MS 39762, USA; 6 University of California, San Diego, La Jolla, CA, 92093 USA; 7 Columbia University, New York, NY 10032 USA; 8 University of Kentucky, Lexington, KY 40506 USA; 9 Emory University, Atlanta, GA 30322, USA

## 

The purpose of this panel is to introduce and describe NIDA's implementation science initiative for justice-involved youth. The goal of JJ-TRIALS is to test implementation strategies for improving the delivery of a continuum of evidence-based substance abuse services as well as improving prevention efforts (for HIV/STDs and substance use disorders) among 36 juvenile justice sites located in Florida, Georgia, Kentucky, Mississippi, New York, Pennsylvania, Texas, and the District of Columbia. The study is scheduled to go into the field July, 2015. The proposed panel will include 3 presentations and a discussant. The first paper describes Aarons' (2011) the Exploration-Preparation-Implementation-Sustainment (EPIS) Model which provides the theoretical framework for the process improvement efforts. The implementation strategies that will be used in this study will also be presented. The second paper described research study design, methods and measures. The third paper describes the diverse settings in which this study will be initiated and will discuss the involvement of juvenile justice partners in the planning of the study protocol. The implications of JJ-TRIALS, including how it fits within NIDA's portfolio, will be highlighted by the discussant.

Using the EPIS model to enhance service delivery in juvenile justice agencies: implementation science in the context of JJ-TRIALS

Juvenile justice (JJ) agencies have long faced service delivery challenges, particularly concerning substance use disorders (SUDs) among their clients. This paper describes the use of Aarons' EPIS model as the implementation science framework governing JJ-TRIALS (Juvenile Justice-Translating Research Interventions for Adolescents in the Legal System). Given SUD prevalence among JJ clients, all youth served by the JJ system should be screened for SUDs, after which they should be referred (as needed) for evidence-based treatment and receive this vital service. JJ-TRIALS seeks to reduce unmet SUD needs by assisting JJ agencies in their efforts to implement best practices and improve service provision along a behavioral health cascade. Each phase of the EPIS model (Aarons, 2011)-Exploration, Preparation, Implementation, and Sustainment-is discussed conceptually and in relation to the JJ-TRIALS project. The Exploration phase features training in behavioral health and Data-Driven Decision Making, after which an agency-identified goal is selected. The Preparation phase consists, among other factors, of decisions to adopt best practices in the pursuit of agency goals and the generation of an agency action plan. The Implementation phase is organized around Plan-Do-Study-Act (PDSA) cycles wherein progress toward agency goals is expected to be exhibited through the application of a series of PDSA cycles conducted by Local Change Teams (LCTs). Finally, the Sustainment phase is characterized by the implementation of key facets of the action plan that were shown to be effective during LCT-led PDSA cycles. The paper concludes by discussing the merits of Aarons' EPIS model in JJ settings when compared with other implementation science paradigms and by reviewing EPIS model adaptations that were necessary to maximize this framework's suitability for use in a JJ context. This project advances the field by addressing how EPIS can be fruitfully applied to enhance service delivery in JJ agencies.

An overview of the JJ-TRIALS study protocol

It is well established that a substantial proportion of adolescent offenders have substance abuse problems, and that linkage to services to identify and treat these problems is often lacking. Successfully linking young offenders (especially those under community supervision) to appropriate and effective services requires several steps that may involve different staff in different agencies. Efforts to reduce unmet behavioral health needs of youth involved in the juvenile justice system requires system-level change. The JJ-TRIALS study targets juvenile justice agencies and the treatment partners to which juveniles are referred in an effort to improve community behavioral health linkages. JJ-TRIALS will compare the effectiveness of two implementation interventions: a Core Intervention, that provides training to staff of juvenile justice agencies on behavioral health needs of juvenile offenders, evidence-based screening, assessment and treatment practices, and Data-Driven Decision Making (DDDM) strategies to promote change across the EPIS phases, versus an Enhanced Intervention, that provides the Core interventions plus support for DDDM through facilitation and implementation teams. A clustered randomized design with a phased roll-out will be used to evaluate the effectiveness of the 2 Intervention arms (direct comparison and comparison of each to baseline). A total of 36 sites representing 7 states and the District of Columbia will be randomized to Core (n = 18) or Enhanced (n = 18) and to one of three start times (to facilitate management by Research Centers). Primary research questions address whether DDDM strategies and facilitation of DDDM tools/implementation teams improve (a) the provision and quality of services along a behavioral health cascade (screening, assessment, referral, and treatment of youth with substance use disorders) and (b) attitudes toward best practices among staff working with justice-involved youth. Exploratory research questions focus on aspects of the implementation process, inter-organizational collaboration, costs associated with each study arm, and youth outcomes.

Community partner involvement in a juvenile justice behavioral health service organizational implementation trial

Each of the six research centers (Columbia University, Emory University, Mississippi State University, Temple University, Texas Christian University, and University of Kentucky) involved in the JJ-TRIALS Cooperative established collaborative partnerships with a state-level juvenile justice agency during the development of their respective proposals. A representative from each of the participating juvenile justice systems committed to participate in the design, planning, and conduct of the research and to attend monthly conference calls and semi-annual in-person meetings of the Cooperative's Steering Committee. The purpose of this presentation is to describe the diverse settings in which the JJ-TRIALS implementation study will be initiated and how partners are involved in designing the study protocol that is feasible for the sites, meets partners' needs, and is scientifically rigorous.

